# Auto-Plastic Operation for the Cure of Ectropium

**Published:** 1845-04

**Authors:** 


*Auto-Plastic Operation for the Cure of Ectropium.*—Dr. Houston exhibited drawings illustrative of a new application of *auto-plasty* to the cure of *ectropium.* The case was that of a young lady, aged fifteen, who, nine years previously, had sustained a burn on the right cheek from a piece of coal, shot out of a blazing fire. The cicatrix extended vertically from the ciliary margin of the lid to opposite the lower part of the nose: transversely, it engaged, at the top, the two internal thirds of the eyelid, and became somewhat narrowed to a point inferiorly. It appeared white and silvery, and was thin in substance, and connected by loose cellular tissue to the parts underneath. The lower eyelid was everted and dragged down, immoveably, in a stroup-like manner, so as to have a great part of its red inner surface exposed. The punctum lacrymale appeared open and unobstructed; but from its mal-position could not take up and carry the tears into the nose. The parts in this region were, as a consequence, excoriated by the trickling of the tears down the cheek. Exposure to cold air, or to a blowing wind, aggravated very much all these inconveniences.

The operation practised by Dr. Houston, in adaptation to this condition of lesion, was as follows:—He made two vertical incisions through the skin, one on each side of the cicatrix, commencing superiorly at the margin of the cartilage, and terminating below, by meeting at an acute angle on the cheek, opposite the ala of the nose. He then dissected up the triangular flap thus formed as far as its attachment to the ciliary margin of the cartilage, and carried the dissection even under this cartilage, so as to break up the adhesion of the latter to the surface of the cheek; which being effected, the lid thus loosened admitted of being pushed up into its proper place—the mucous membrane lying against the globe of the eye, and the surface, which hitherto lay on the cheek, being now turned forwards and covered by the flap of the cicatrix. By this step, as may readily be understood, a perfect lid, with mucous membrane inside and skin outside, was constructed out of original, although imperfect, materials, capable f being raised and depressed upon the eye, and of permitting the lacrymal punctum on its margin to come into apposition with its fellow of the upper lid. The next step in the operation consisted in covering over the space on the cheek left vacant by the displacement of the flap, and which now looked doubly large from the retraction of the edges of the wound. To effect this, the incised margins of the integuments were dissected up for a distance sufficient to render them pliable, and then brought together in a vertical line over the centre of the wound, in which position they were fixed by a fine hare-lip needle at the top, and a single thread of suture near the bottom, in a manner calculated to favour union by the first intention. The parts thus adjusted, lying accurately and easily in position and exhibiting an appearance perfectly free from deformity, were farther supported by adhesive plaster and bandage. Strict rest was enjoined.

At the expiration of thirty-six hours the threads were cut across, without, however, removing the needle or stitch from their places. The wound appeared quite dry, and the lips lay, united, accurately together. There was no pain, and very little œdema. The eversion had been completely removed, and the free edge of the lower lid lay straight and on the same line with that of the upper. At the end of forty-eight hours both needle and suture were withdrawn, the union appearing perfect in every part. At this period, the patient feeling no uneasiness, and, perhaps, over elated at the prospect of improvement in her beauty, indulged rather much in moving about, laughing, and talking with her friends, whereby, as occurring before union was firmly established, a slight excess of inflammatory action was superinduced. In seventy-two hours the dressings were found stained with pus, and the lips of the wound loosened. There was, however, but little separation, as the flaps, having been fixed in the right place by the previous adhesive inflammation, did not retract them much. A bread and water poultice was applied for a night; after which the inflammation passed off, and the wound soon healed by a narrow line of granulations in a manner to fulfil every desirable indication. The red surface of the lid had ceased to be visible externally; the tears no longer trinkled down the cheek; and, although a trifling mark remained to show the point of healing by granulation, and which, certainly, would not have existed but for the accident of the secondary inflammation, still the relief from deformity (as shown by the drawing) was nearly complete. It might be supposed that the skin of the cicatrix, pushed up, as above described, against the cartilage of the lower lid, would have constituted a bulky prominence there; but such was not the fact, as only a thin layer had been removed for transplantation, and, as even that had shrunk away much during and subsequent to the healing of the parts. Dr. Houston said that it appeared unnecessary for him to enumerate the other modes of operating for ectropium, in order that the difference between them and the mode here practised should be appreciated. That difference would be obvious to every one. Here there had been no cutting away of any part. The skin of the cicatrix was transplanted so as to form an outer integument for the eyelid, and to prop up that lid against the globe; whilst the skin of the cheek, brought in from the sides, was so applied as not only to cover over the part deserted by the cicatrix, but, by taking its place, to prevent the latter from again descending in such a manner as to contribute a second time to the production of the ectropium.—*Dublin Medical Press.*

